# 2,3-Diamino­pyridinium hydrogen malonate

**DOI:** 10.1107/S1600536812050386

**Published:** 2012-12-15

**Authors:** Kaliyaperumal Thanigaimani, Nuridayanti Che Khalib, Suhana Arshad, Ibrahim Abdul Razak

**Affiliations:** a School of Physics, Universiti Sains Malaysia, 11800 USM, Penang, Malaysia

## Abstract

In the title mol­ecular salt, C_5_H_8_N_3_^+^·C_3_H_3_O_4_^−^, the cation is essentially planar, with a maximum deviation of 0.005 (1) Å for all non-H atoms. In the anion, an intra­molecular O—H⋯O hydrogen bond generates an *S*(6) ring. In the crystal, the cations and anions are connected *via* N—H⋯O hydrogen bonds and a weak C—H⋯O inter­action, forming layers parallel to the *ab* plane.

## Related literature

For backgroup to the chemistry of substituted pyridines, see: Amr *et al.* (2006 ▶); Bart *et al.* (2001 ▶); Shinkai *et al.* (2000 ▶). For related structures, see: Betz *et al.* (2011 ▶); Hemamalini *et al.* (2011 ▶); Balasubramani & Fun (2009 ▶); Fun & Balasubramani (2009 ▶). For the conformation of the malonate ion, see: Djinović *et al.* (1990 ▶). For hydrogen-bond motifs, see: Bernstein *et al.* (1995 ▶). For bond-length data, see: Allen *et al.* (1987 ▶). For stability of the temperature controller used for the data collection, see: Cosier & Glazer (1986 ▶).
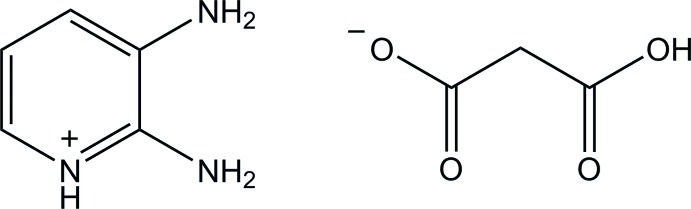


## Experimental

### 

#### Crystal data

C_5_H_8_N_3_^+^·C_3_H_3_O_4_^−^
*M* *_r_* = 213.20
Monoclinic, 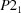

*a* = 5.0843 (1) Å
*b* = 8.0771 (1) Å
*c* = 11.1928 (2) Å
β = 91.214 (1)°
*V* = 459.55 (1) Å^3^
*Z* = 2
Mo *K*α radiation
μ = 0.13 mm^−1^
*T* = 100 K
0.28 × 0.25 × 0.14 mm

#### Data collection

Bruker SMART APEXII CCD area-detector diffractometer
Absorption correction: multi-scan (*SADABS*; Bruker, 2009 ▶) *T*_min_ = 0.966, *T*_max_ = 0.983
6631 measured reflections
1778 independent reflections
1695 reflections with *I* > 2σ(*I*)
*R*_int_ = 0.024

#### Refinement

*R*[*F* ^2^ > 2σ(*F* ^2^)] = 0.037
*wR*(*F* ^2^) = 0.094
*S* = 1.07
1778 reflections
160 parameters
1 restraint
H atoms treated by a mixture of independent and constrained refinement
Δρ_max_ = 0.35 e Å^−3^
Δρ_min_ = −0.26 e Å^−3^

### 

Data collection: *APEX2* (Bruker, 2009 ▶); cell refinement: *SAINT* (Bruker, 2009 ▶); data reduction: *SAINT*; program(s) used to solve structure: *SHELXTL* (Sheldrick, 2008 ▶); program(s) used to refine structure: *SHELXTL*; molecular graphics: *SHELXTL*; software used to prepare material for publication: *SHELXTL* and *PLATON* (Spek, 2009 ▶).

## Supplementary Material

Crystal structure: contains datablock(s) global, I. DOI: 10.1107/S1600536812050386/is5228sup1.cif

Structure factors: contains datablock(s) I. DOI: 10.1107/S1600536812050386/is5228Isup2.hkl

Supplementary material file. DOI: 10.1107/S1600536812050386/is5228Isup3.cml

Additional supplementary materials:  crystallographic information; 3D view; checkCIF report

## Figures and Tables

**Table 1:** Hydrogen-bond geometry (Å, °)

*D*—H⋯*A*	*D*—H	H⋯*A*	*D*⋯*A*	*D*—H⋯*A*
O3—H1*O*3⋯O1	0.93 (4)	1.63 (3)	2.5208 (16)	159 (3)
N3—H2*N*3⋯O4^i^	0.88 (3)	2.16 (3)	2.9133 (19)	143 (2)
N2—H2*N*2⋯O2^ii^	0.87 (3)	2.15 (3)	3.0066 (18)	168 (2)
N1—H1*N*1⋯O1^iii^	0.92 (3)	1.87 (3)	2.7782 (16)	168 (3)
N2—H1*N*2⋯O2^iii^	0.88 (3)	2.12 (3)	2.9470 (18)	157 (3)
N3—H1*N*3⋯O2^ii^	0.87 (2)	2.18 (2)	3.0574 (19)	178 (3)
C7—H7*B*⋯O2^iv^	0.99	2.46	3.3532 (19)	149

Symmetry codes: (i) 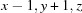; (ii) 
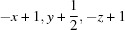
; (iii) 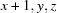; (iv) 
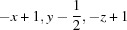
.

**Fig. 1.**
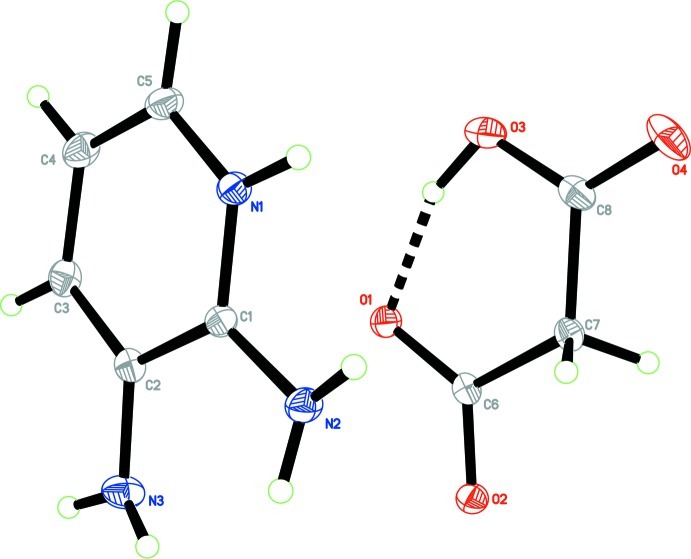
The molecular structure of the title compound with atom labels with 50% probability displacement ellipsoids.

**Fig. 2.**
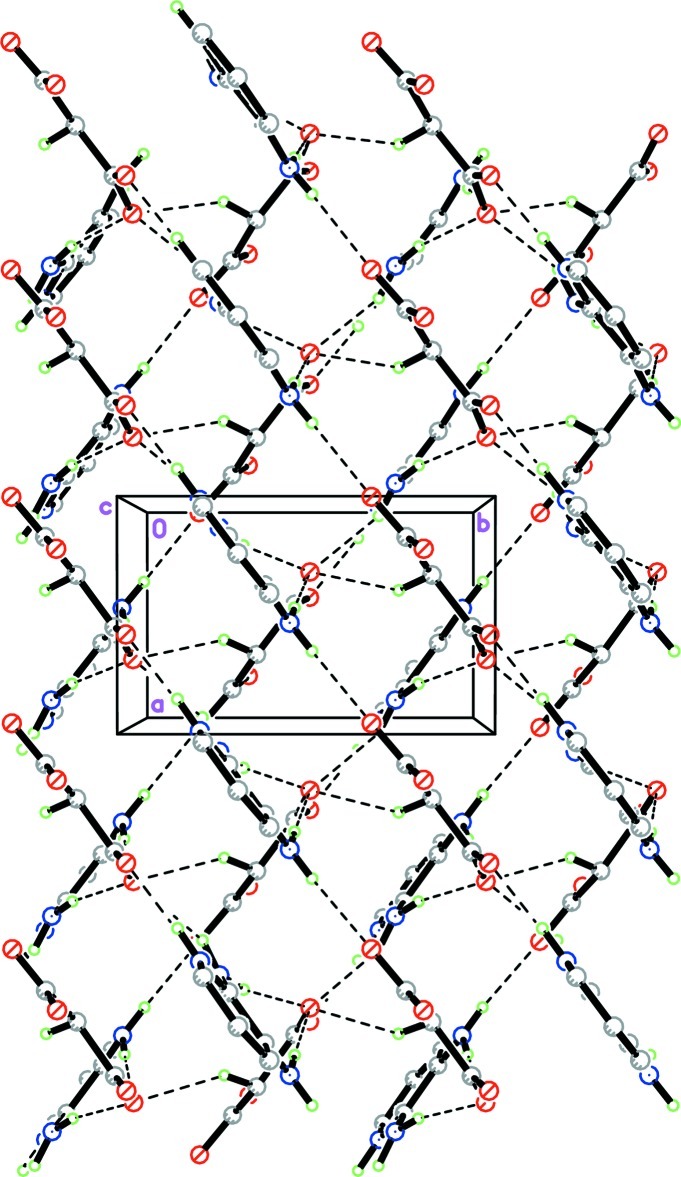
The crystal packing of the title compound. The H atoms not involved in the intermolecular interactions (dashed lines) have been omitted for clarity.

## supplementary crystallographic information

### Comment

Pyridine and its derivatives continue to attract great interest due to the wide variety of interesting biological activities observed for these compounds, such as anticancer, analgesic, antimicrobial and antidepressant activities (Amr *et al.*, 2006; Bart *et al.*, 2001; Shinkai *et al.*, 2000). They are also often involved in hydrogen-bond interactions. The related crystal structures of 2,3-diaminopyridinium 2-hydroxybenzoate (Hemamalini *et al.*, 2011), 2,3-diaminopyrimidinium benzoate (Balasubramani & Fun, 2009) and 2,3-diaminopyridinium 4-hydroxybenzoate (Fun & Balasubramani, 2009) have been recently reported. In order to study potential hydrogen bonding interactions, the crystal structure determination of the title compound (I) was carried out.

The asymmetric unit (Fig. 1) contains one 2,3-Diaminopyridinium cation and one hydrogen malonate anion. The proton transfers from one of the carboxyl group oxygen atom (O1) to atom N1 of 2,3-diaminopyridine resulted in widening of C1—N1—C5 angle of the pyridinium ring to 123.69 (13)°, compared to the corresponding angle of 118.97 (15)° in neutral 2,3-diaminopyridine (Betz *et al.*, 2011). The 2,4-diaminopyrinium cation is planar, with a maximum deviation of 0.005 (1) Å for atom N1. The bond lengths (Allen *et al.*, 1987) and angles are normal.

In the crystal packing (Fig. 2), the protonated N1 atom and the 2-amino group (N2) is hydrogen-bonded to the carboxylate oxygen atoms (O1 and O2) *via* a pair of intermolecular N1—H1N1···O1^iii^ and N2—H1N2···O2^iii^ hydrogen bonds (symmetry code in Table 1), forming a ring motif *R*_2_^2^(8) (Bernstein *et al.*, 1995). Atom O3 of the carboxyl group of the hydrogen malonate anion forms an intramolecular O3—H1O3···O1 hydrogen bond with the O atom of the carboxylate group (O1) [with graph-set notation S(6)], leading to a folded conformation. A similar intramolecular hydrogen bond has been observed in the crystal structures of benzylammonium hydrogen malonate and 4-picolinium hydrogen malonate (Djinović *et al.*, 1990). The 2-amino groups (N2 and N3) are involved in the intermolecular N—H···O hydrogen bonds with hydrogen malonate oxygen atom (O2), forming an *R*_2_^1^(7) ring motif. The crystal structure is further stabilized by a weak C7—H7B···O2^iv^ interaction (symmetry code in Table 1), forming a layer lying parallel to the *ab* plane.

### Experimental

Hot methanol solutions (20 ml) of 2,3-diaminopyrimidine (27 mg, Aldrich) and malonic acid (26 mg, Merck) were mixed and warmed over a heating magnetic stirrer hotplate for a few minutes. The resulting solution was allowed to cool slowly at room temperature and crystals of the title compound (I) appeared after a few days.

### Refinement

O- and N-bound H atoms were located in a difference Fourier map and allowed to be refined freely [O—H = 0.93 (4) Å and N—H = 0.87 (3)–0.92 (3) Å]. The remaining hydrogen atoms were positioned geometrically (C—H = 0.95 or 0.99 Å) and refined using a riding model, with *U*_iso_(H) = 1.2*U*_eq_(C). In the final refinement, 1237 Friedel pairs were merged.

### Crystal data

**Table:**

C_5_H_8_N_3_^+^·C_3_H_3_O_4_^−^	*F*(000) = 224
*M**_r_* = 213.20	*D*_x_ = 1.541 Mg m^−^^3^
Monoclinic, *P*2_1_	Mo *K*α radiation, λ = 0.71073 Å
Hall symbol: P 2yb	Cell parameters from 3153 reflections
*a* = 5.0843 (1) Å	θ = 3.1–32.6°
*b* = 8.0771 (1) Å	µ = 0.13 mm^−^^1^
*c* = 11.1928 (2) Å	*T* = 100 K
β = 91.214 (1)°	Block, brown
*V* = 459.55 (1) Å^3^	0.28 × 0.25 × 0.14 mm
*Z* = 2	

### Data collection

**Table:**

Bruker SMART APEXII CCD area-detector diffractometer	1778 independent reflections
Radiation source: fine-focus sealed tube	1695 reflections with *I* > 2σ(*I*)
Graphite monochromator	*R*_int_ = 0.024
φ and ω scans	θ_max_ = 32.7°, θ_min_ = 1.8°
Absorption correction: multi-scan (*SADABS*; Bruker, 2009)	*h* = −7→7
*T*_min_ = 0.966, *T*_max_ = 0.983	*k* = −10→12
6631 measured reflections	*l* = −17→16

### Refinement

**Table:**

Refinement on *F*^2^	Primary atom site location: structure-invariant direct methods
Least-squares matrix: full	Secondary atom site location: difference Fourier map
*R*[*F*^2^ > 2σ(*F*^2^)] = 0.037	Hydrogen site location: inferred from neighbouring sites
*wR*(*F*^2^) = 0.094	H atoms treated by a mixture of independent and constrained refinement
*S* = 1.07	*w* = 1/[σ^2^(*F*_o_^2^) + (0.0564*P*)^2^ + 0.057*P*] where *P* = (*F*_o_^2^ + 2*F*_c_^2^)/3
1778 reflections	(Δ/σ)_max_ < 0.001
160 parameters	Δρ_max_ = 0.35 e Å^−^^3^
1 restraint	Δρ_min_ = −0.26 e Å^−^^3^

### Special details

**Table:**

Experimental. The crystal was placed in the cold stream of an Oxford Cryosystems Cobra open-flow nitrogen cryostat (Cosier & Glazer, 1986) operating at 100.0 (1) K.
Geometry. All e.s.d.'s (except the e.s.d. in the dihedral angle between two l.s. planes) are estimated using the full covariance matrix. The cell e.s.d.'s are taken into account individually in the estimation of e.s.d.'s in distances, angles and torsion angles; correlations between e.s.d.'s in cell parameters are only used when they are defined by crystal symmetry. An approximate (isotropic) treatment of cell e.s.d.'s is used for estimating e.s.d.'s involving l.s. planes.
Refinement. Refinement of *F*^2^ against ALL reflections. The weighted *R*-factor *wR* and goodness of fit *S* are based on *F*^2^, conventional *R*-factors *R* are based on *F*, with *F* set to zero for negative *F*^2^. The threshold expression of *F*^2^ > σ(*F*^2^) is used only for calculating *R*-factors(gt) *etc*. and is not relevant to the choice of reflections for refinement. *R*-factors based on *F*^2^ are statistically about twice as large as those based on *F*, and *R*- factors based on ALL data will be even larger.

### Fractional atomic coordinates and isotropic or equivalent isotropic displacement parameters (Å^2^)

**Table:**

	*x*	*y*	*z*	*U*_iso_*/*U*_eq_	
O1	0.4162 (2)	0.50089 (15)	0.24888 (10)	0.0139 (2)	
O2	0.3017 (2)	0.50412 (15)	0.44060 (10)	0.0139 (2)	
O3	0.7878 (2)	0.31758 (17)	0.18357 (11)	0.0209 (3)	
O4	1.0228 (2)	0.17757 (17)	0.31863 (13)	0.0222 (3)	
N1	0.9986 (2)	0.70786 (17)	0.18711 (12)	0.0123 (2)	
N2	0.8913 (3)	0.75149 (17)	0.38452 (12)	0.0136 (3)	
N3	0.4668 (3)	0.95043 (19)	0.30805 (14)	0.0163 (3)	
C1	0.8441 (3)	0.78141 (18)	0.26789 (13)	0.0107 (3)	
C2	0.6311 (3)	0.88272 (18)	0.22541 (14)	0.0119 (3)	
C3	0.5946 (3)	0.8991 (2)	0.10331 (14)	0.0142 (3)	
H3A	0.4551	0.9663	0.0730	0.017*	
C4	0.7607 (3)	0.8179 (2)	0.02293 (14)	0.0161 (3)	
H4A	0.7326	0.8294	−0.0608	0.019*	
C5	0.9617 (3)	0.7228 (2)	0.06655 (14)	0.0153 (3)	
H5A	1.0754	0.6673	0.0135	0.018*	
C6	0.4477 (3)	0.45888 (19)	0.35774 (14)	0.0112 (3)	
C7	0.6803 (3)	0.34858 (19)	0.39221 (14)	0.0129 (3)	
H7A	0.7997	0.4142	0.4448	0.016*	
H7B	0.6122	0.2564	0.4410	0.016*	
C8	0.8449 (3)	0.27351 (19)	0.29470 (15)	0.0148 (3)	
H2N3	0.343 (5)	1.013 (4)	0.276 (2)	0.023 (6)*	
H2N2	0.814 (5)	0.817 (4)	0.434 (2)	0.026 (6)*	
H1N1	1.136 (6)	0.645 (4)	0.218 (3)	0.044 (8)*	
H1N2	1.043 (5)	0.703 (4)	0.401 (2)	0.029 (6)*	
H1N3	0.534 (5)	0.963 (3)	0.380 (2)	0.018 (5)*	
H1O3	0.654 (6)	0.395 (5)	0.190 (3)	0.048 (9)*	

### Atomic displacement parameters (Å^2^)

**Table:**

	*U*^11^	*U*^22^	*U*^33^	*U*^12^	*U*^13^	*U*^23^
O1	0.0137 (4)	0.0153 (5)	0.0126 (5)	0.0024 (4)	−0.0005 (4)	0.0011 (4)
O2	0.0129 (4)	0.0152 (5)	0.0136 (5)	0.0025 (4)	0.0018 (3)	0.0004 (4)
O3	0.0217 (5)	0.0231 (6)	0.0183 (6)	0.0068 (5)	0.0060 (4)	−0.0012 (5)
O4	0.0143 (5)	0.0164 (6)	0.0360 (8)	0.0056 (4)	0.0016 (4)	−0.0008 (5)
N1	0.0115 (5)	0.0135 (6)	0.0119 (6)	0.0010 (4)	0.0004 (4)	0.0006 (5)
N2	0.0134 (5)	0.0160 (6)	0.0114 (6)	0.0024 (4)	−0.0001 (4)	0.0004 (5)
N3	0.0115 (5)	0.0211 (6)	0.0162 (6)	0.0053 (5)	0.0001 (4)	−0.0023 (5)
C1	0.0096 (5)	0.0114 (6)	0.0112 (6)	−0.0005 (4)	0.0006 (4)	0.0001 (5)
C2	0.0099 (5)	0.0112 (6)	0.0147 (7)	−0.0001 (5)	−0.0005 (4)	0.0005 (5)
C3	0.0124 (5)	0.0154 (6)	0.0148 (7)	0.0012 (5)	−0.0022 (5)	0.0026 (6)
C4	0.0168 (6)	0.0201 (7)	0.0114 (7)	0.0012 (5)	−0.0011 (5)	0.0021 (6)
C5	0.0165 (6)	0.0184 (7)	0.0111 (6)	0.0015 (6)	0.0022 (5)	0.0002 (6)
C6	0.0089 (5)	0.0101 (6)	0.0145 (6)	−0.0006 (5)	−0.0007 (4)	0.0002 (5)
C7	0.0118 (5)	0.0130 (6)	0.0140 (6)	0.0028 (5)	−0.0007 (4)	0.0012 (5)
C8	0.0113 (6)	0.0114 (6)	0.0219 (8)	−0.0008 (5)	0.0032 (5)	−0.0019 (6)

### Geometric parameters (Å, º)

**Table:**

O1—C6	1.2716 (19)	N3—H1N3	0.87 (2)
O2—C6	1.2542 (17)	C1—C2	1.4305 (19)
O3—C8	1.320 (2)	C2—C3	1.382 (2)
O3—H1O3	0.93 (4)	C3—C4	1.409 (2)
O4—C8	1.2168 (19)	C3—H3A	0.9500
N1—C1	1.3482 (18)	C4—C5	1.361 (2)
N1—C5	1.3638 (19)	C4—H4A	0.9500
N1—H1N1	0.92 (3)	C5—H5A	0.9500
N2—C1	1.344 (2)	C6—C7	1.524 (2)
N2—H2N2	0.87 (3)	C7—C8	1.516 (2)
N2—H1N2	0.88 (3)	C7—H7A	0.9900
N3—C2	1.3731 (19)	C7—H7B	0.9900
N3—H2N3	0.88 (3)		
			
C8—O3—H1O3	104.9 (19)	C5—C4—C3	119.31 (14)
C1—N1—C5	123.70 (13)	C5—C4—H4A	120.3
C1—N1—H1N1	116.0 (18)	C3—C4—H4A	120.3
C5—N1—H1N1	120.3 (18)	C4—C5—N1	119.42 (14)
C1—N2—H2N2	115.8 (16)	C4—C5—H5A	120.3
C1—N2—H1N2	114.6 (16)	N1—C5—H5A	120.3
H2N2—N2—H1N2	123 (2)	O2—C6—O1	124.51 (13)
C2—N3—H2N3	113.0 (16)	O2—C6—C7	116.82 (13)
C2—N3—H1N3	115.7 (15)	O1—C6—C7	118.66 (12)
H2N3—N3—H1N3	125 (2)	C8—C7—C6	119.27 (13)
N2—C1—N1	118.52 (13)	C8—C7—H7A	107.5
N2—C1—C2	122.95 (13)	C6—C7—H7A	107.5
N1—C1—C2	118.48 (13)	C8—C7—H7B	107.5
N3—C2—C3	123.85 (13)	C6—C7—H7B	107.5
N3—C2—C1	118.10 (13)	H7A—C7—H7B	107.0
C3—C2—C1	117.95 (12)	O4—C8—O3	121.78 (15)
C2—C3—C4	121.14 (13)	O4—C8—C7	121.03 (16)
C2—C3—H3A	119.4	O3—C8—C7	117.19 (13)
C4—C3—H3A	119.4		
			
C5—N1—C1—N2	176.82 (14)	C2—C3—C4—C5	−0.5 (2)
C5—N1—C1—C2	−0.8 (2)	C3—C4—C5—N1	0.0 (2)
N2—C1—C2—N3	−0.7 (2)	C1—N1—C5—C4	0.7 (2)
N1—C1—C2—N3	176.80 (14)	O2—C6—C7—C8	172.90 (13)
N2—C1—C2—C3	−177.26 (15)	O1—C6—C7—C8	−8.2 (2)
N1—C1—C2—C3	0.3 (2)	C6—C7—C8—O4	−175.86 (14)
N3—C2—C3—C4	−175.92 (16)	C6—C7—C8—O3	4.6 (2)
C1—C2—C3—C4	0.4 (2)		

### Hydrogen-bond geometry (Å, º)

**Table:**

*D*—H···*A*	*D*—H	H···*A*	*D*···*A*	*D*—H···*A*
O3—H1*O*3···O1	0.93 (4)	1.63 (3)	2.5208 (16)	159 (3)
N3—H2*N*3···O4^i^	0.88 (3)	2.16 (3)	2.9133 (19)	143 (2)
N2—H2*N*2···O2^ii^	0.87 (3)	2.15 (3)	3.0066 (18)	168 (2)
N1—H1*N*1···O1^iii^	0.92 (3)	1.87 (3)	2.7782 (16)	168 (3)
N2—H1*N*2···O2^iii^	0.88 (3)	2.12 (3)	2.9470 (18)	157 (3)
N3—H1*N*3···O2^ii^	0.87 (2)	2.18 (2)	3.0574 (19)	178 (3)
C7—H7*B*···O2^iv^	0.99	2.46	3.3532 (19)	149

Symmetry codes: (i) *x*−1, *y*+1, *z*; (ii) −*x*+1, *y*+1/2, −*z*+1; (iii) *x*+1, *y*, *z*; (iv) −*x*+1, *y*−1/2, −*z*+1.
